# Daily snuff use during pregnancy, gestational length and birth weight; register-based study

**DOI:** 10.1186/s12884-024-06863-8

**Published:** 2024-10-04

**Authors:** Rolv T. Lie, Maria C. Magnus, Håkon K. Gjessing, Allen J. Wilcox, Siri E. Håberg

**Affiliations:** 1 Department of Global Public Health and Primary Care, University of Bergen, Bergen, Norway; 2 Centre for Fertility and Health, Norwegian Institute of Public Health, Oslo, Norway; 3 Epidemiology Branch, National Institute of Environmental Health Sciences, Durham, NC USA

**Keywords:** Smokeless tobacco, Maternal snuff use, Maternal smoking, Pregnancy, Gestational length, Birth weight

## Abstract

**Background:**

Snuff is a smokeless source of nicotine that is common in Scandinavia and increasingly used by women of fertile age. Persistent use of snuff during pregnancy has been associated with adverse pregnancy outcomes. Emerging data from the Medical Birth Registry of Norway distinguishes between occasional use and daily use. We provide preliminary estimates of associations between frequency of snuff and gestational length and birth weight.

**Methods:**

Data on snuff use during pregnancies delivered in 2020 and 2021 were available for the west and central regions of Norway. Associations of snuff use with gestational length and birth weight at term (39–41 weeks) were estimated using quantile regression at the 25th, the 50th and the 75th percentiles, with adjustments for mother’s age, pre-pregnancy weight, and parity. We compared associations with the pregnancy outcomes according to maternal snuff and cigarette use.

**Results:**

12.4% of 18 042 non-smoking women reported daily use of snuff before pregnancy, and 4.6% reported continuing use during pregnancy, with 1.2% still reporting daily use in the last trimester. Women with daily use through the last trimester delivered babies with a median gestational length reduced by 3.4 days (95% CI: -5.0 to -1.7 days) compared with women who never used snuff. The reduction was even stronger at the 25th percentile of gestational age. The median term birth weight was reduced by 44 g (95% CI: -134 to 46 g). These associations were much weaker for women who quit snuff at some point during pregnancy or used snuff only occasionally. Mothers who smoked daily through the last trimester had a median gestational length reduced by 2.1 days (95% CI: -2.7 to -1.4) and a median term birth weight reduced by 294 g (95% CI: -325 to -262) compared with never-smokers.

**Conclusions:**

Daily snuff use through the last trimester reduced the median gestational length by more than three days. Snuff reduced birth weight, but not as much as smoking, suggesting that the predominant effect of smoking on fetal growth is not through nicotine but through the additional toxic chemicals in cigarettes or by reduced oxygen supply to the fetus.

**Supplementary Information:**

The online version contains supplementary material available at 10.1186/s12884-024-06863-8.

## Background

Tobacco smoke contains a complex mixture of toxic chemicals that are hazards to health [1]. Smoking during pregnancy is well established as a cause of still birth, preterm birth, and reduced birth weight [2, 3]. In recent decades, smoking has become less common in western countries and alternative sources of nicotine have become more popular [4]. In the Scandinavian countries, Swedish snus, or snuff, is increasingly used (Figure S1, supplementary material). According to 2021 data from Statistics Norway, 17% of Norwegian women between 25 and 34 years of age report daily use of snuff [5]. Snuff does not contain toxic combustion products and may be perceived as a safer source of nicotine [6, 7]. However, nicotine is highly addictive and regular use of snuff may result in persistently high levels of nicotine in the maternal blood during pregnancy [8].

The Swedish Medical Birth Registry has been a prime source of studies on snuff use in pregnancy. Studies using this data source have consistently found that snuff use throughout pregnancy is associated with moderately increased risks of fetal death, preterm delivery, and reduced birth weight. These Swedish studies have provided much of the published data for a recent meta-analysis and review [9]. The Swedish data, however, do not include information on frequency of use and can’t distinguish between occasional and regular use [10].

The Medical Birth Registry of Norway (MBRN) recently started collection of snuff exposure before and during pregnancy. These Norwegian data have the advantage of distinguishing between daily and occasional snuff use. Data on snuff will be available for all births in Norway once data collection systems are updated in all regions. As of today, data are available from two health regions in the western and central parts of Norway.

We performed an analysis of data from 2020 and 2021 to provide preliminary estimates of the association between snuff use during pregnancy and two outcomes: length of pregnancy and birth weight. We distinguish between daily use and occasional snuff use during distinct time periods of pregnancy – before pregnancy (early quitters), only up to the 1st trimester (late quitters) and throughout the last trimester. We also compared snuff and cigarette use in their associations with pregnancy outcomes.

## Methods

Information on all births in Norway has routinely been collected by the Medical Birth Registry of Norway since 1967 [11]. The newest update of the data collection system includes information on snuff use in pregnancy. The regional health administrations of Helse Vest (covering the western part of Norway) and Helse Midt (covering the central part) updated to the newest version in 2020, and the rest of the country is expected to follow. Smoking information has been available in the birth registry for all Norwegian mothers since 2000.

**Fig. 1:**
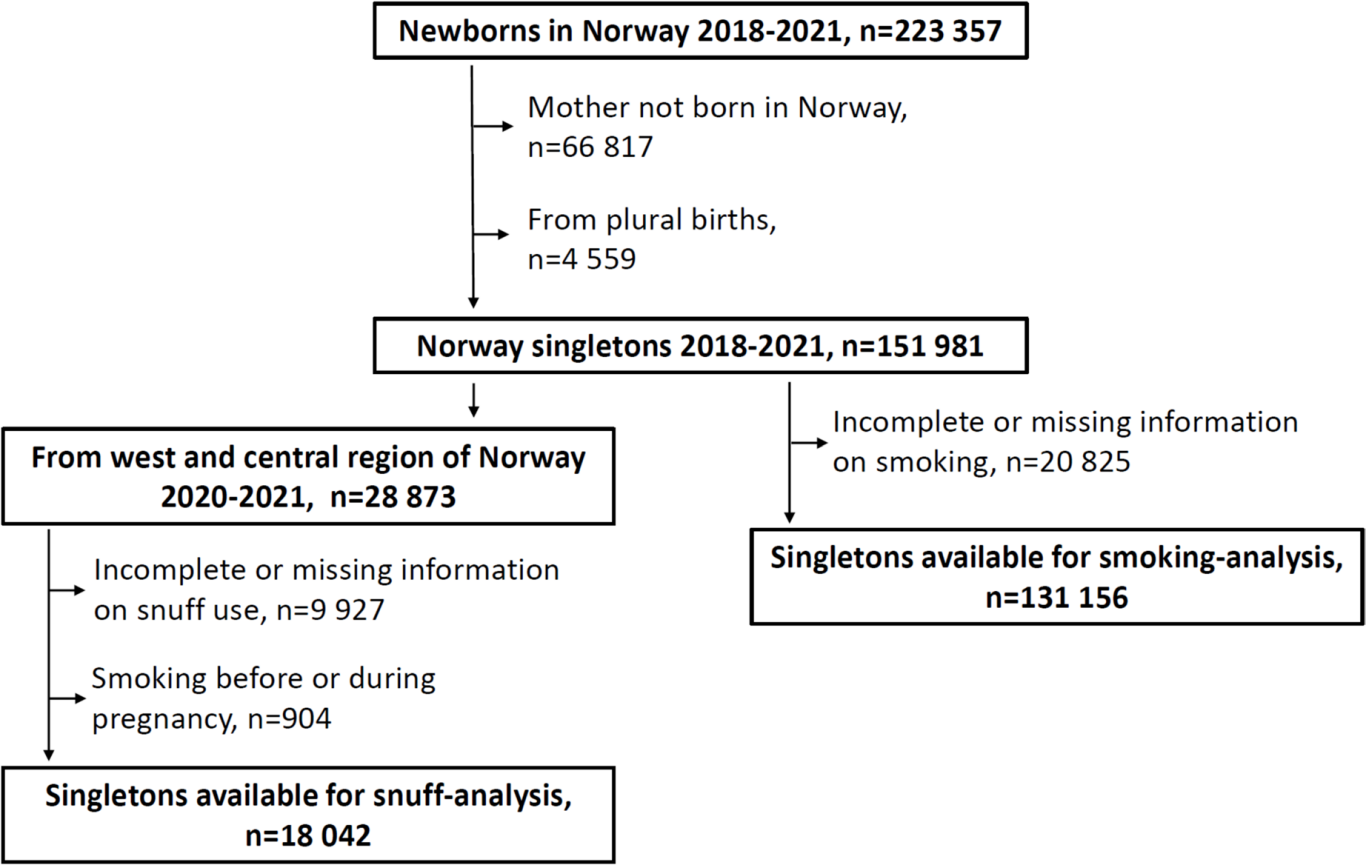
Procedure of extraction of data for the snuff analysis and the smoking analysis from the Medical Birth Registry of Norway 2018–2021. Data for the snuff analysis was only available for 2020–2021 for the regions of west and central Norway

To facilitate the analysis of both snuff use and smoking, we identified 223 357 newborns in Norway in the period 2018 to 2021 (Fig. 1). We excluded 66 817 newborns with mothers born outside Norway and 4 559 newborns from plural births. From the remaining 151 981 singletons, we identified 28 873 born in the western and central regions of Norway for our snuff analysis. 9 927 were excluded with missing information on snuff use and 904 were excluded because the mother reported smoking before or during the pregnancy. The remaining 18 042 newborns were the basis for the snuff analysis. From the 151 981 singletons in the period 2018–2021 we then excluded 20 825 newborns with missing or incomplete information on maternal smoking to obtain 131 156 singletons for our smoking analysis. We were not able to exclude snuff use in the smoking analysis since snuff use was not recorded for the extended time-period and geographical area that was used to obtain improved precision in the smoking analysis. While snuff use has increased, daily smoking has shown a strong decline in Norway [5, 12].

Data on snuff use in the MBRN is derived partly from data collected prospectively at the antenatal visits, and partly from interview with the woman upon arrival at the birth clinic. Women had to provide specific informed consent for collection of these data, and the lack of recorded informed consent contributed to a high number with missing information. Data on snuff use is collected for three time points: before pregnancy, during the first trimester, and during the last trimester. For each time point there are four possible exposure categories: “no use,” “used snuff sometimes,” “used snuff daily,” and “unknown frequency of use.” We pooled “unknown frequency” and “used snuff sometimes” into a category of “occasional use,” creating a three-level dose variable: 1 (no snuff use), 2 (occasional use), and 3 (daily use). This was done for each of the three time periods of exposure (before pregnancy, during the first trimester and during the last trimester).

**Fig. 2:**
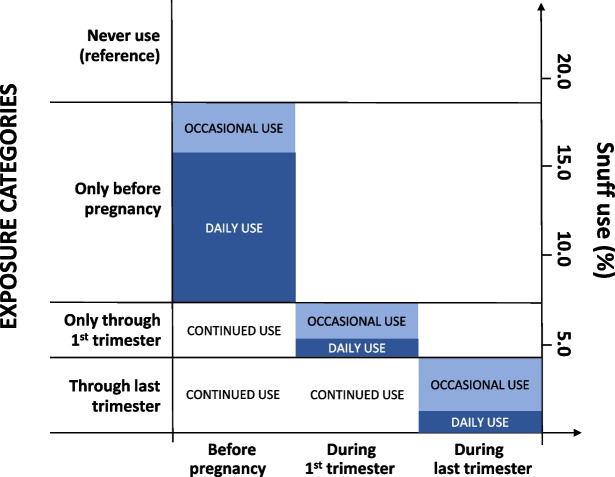
Definition of exposure categories and frequency of daily and occasional snuff use are shown for three time points related to pregnancy. Blue and light blue bars show frequency of daily users and occasional users who quit at each time point. These categories of users at each time point are used as exposure categories. Never-users are used as reference category. Occasional use includes reported use of snuff sometimes or with unknown frequency

We then created one common exposure variable with never-use as the reference category (Fig. 2). Occasional and daily use only before pregnancy were coded as exposure categories 1 and 2, occasional use and daily use only though 1st trimester were coded as exposure categories 3 and 4, while occasional use and daily use during last trimester were coded as exposure categories 5 and 6. Very few women initiated or increased their use of snuff during pregnancy, so that their latest period of usage can be assumed to include some use up to that point in time.

Smoking variables in the birth registry are based on the same three time-points and same categorization of dose as the snuff variables. Again, we pooled the category of unknown frequency with the category of occasional use, which produced a three-level dose-variable: 1. no smoking, 2, occasional smoking, and 3, daily smoking. Smokers were then categorized into exposure categories similar to those for snuff use (Fig. 2) according to the latest period of pregnancy in which they were exposed and with never-smokers as a common reference category.

The gestational length variable in the MBRN was estimated from the ultrasound term estimate for more than 95% of the births in 2020. When ultrasound information was lacking, the last menstrual period was used to estimate gestational length. Norway has a uniform system of weighing newborns at the hospital after birth and recording of the birth weight is mandatory in the MBRN for all newborns, including stillbirths and small babies.

### Statistical analysis

Our main method of analysis was quantile regression (specifically regressions on the 25th, 50th, and 75th quantiles), with adjustments for potential confounders (mother’s age, parity, and mother’s weight before the pregnancy). The associations of exposures with outcomes can be assessed separately for the lower, central, and upper parts of the distribution of gestational length and birth weight. Similar analyses were performed for smoking, and results were compared with the snuff analyses. Differences in association estimates for snuff use and smoking were tested with Wald tests.

To reduce variations in birth weight caused by gestational length, we restricted our analyses of birth weight to births occurring in the narrow term time-window between the 39th and the 41st week of gestation, which comprised 73.5% of all births. We also stratified deliveries into spontaneous onset of labor versus non-spontaneous (induced labor or cesarean section).

To allow inspection of associations along the continuum of the distributions, we also graphed the cumulative distribution functions of gestational length and birth weight for exposure in the last trimester. All analyses were performed with STATA v. 17 (StataCorp LLC, 4905 Lakeway Drive, College Station, Texas 77,845, USA).

## Results

Very few daily snuff users were excluded because they also smoked (Table S1, supplementary material). Among the 18 042 non-smoking women included in our snuff analysis, 12.4% used snuff daily before their pregnancy and 4.3% used snuff occasionally (Fig. 2). Two-thirds of daily users (8.4% of all women) quit snuff use before pregnancy, while about half of the occasional users (1.8%) quit. A total of 2.5% of all women reported daily snuff use in the first trimester, while 2.8% reported occasional use. The great majority of these had also used snuff before the pregnancy. In the last trimester 1.2% of all women reported daily snuff use while 3.0% reported occasional use. 94% of women who reported daily snuff use in the last trimester also reported daily snuff use before pregnancy and in the first trimester.

Table 1 shows the mean and standard deviation of gestational length and birth weight by categories of snuff use in the last trimester. More frequent use of snuff was associated with greater reductions of both mean gestational length and birth weight. Birth weight was less affected among the term births. Table 1 also shows associations between maternal characteristics and snuff use. Daily snuff use was higher among younger women, women of higher parity, and women with heavier pre-pregnancy weight. Similar information for women who were smoking in pregnancy is shown in Table S2 (supplementary material). Larger differences are apparent for birth weight with smoking than with snuff use. The distributions of smoking and snuff use are similar across categories of maternal characteristics except that smoking was more common among older women.

**Table 1:** Descriptive information of key variables by categories of snuff use, west and central region of Norway, 2020–2021

		**Snuff use in last trimester**			**Snuff**	
		**Daily use**	**Occasional use**	**No use**	**never-use**	**Total**
		Mean (SD)	Mean (SD)	Mean (SD)	Mean (SD)	Mean (SD)
**Gestational length**	(days)	274.3 (16.8)	275.9 (18.6)	278.5 (13.3)	278.4 (13.6)	278.4 (13.6)
**Birth weight**^a^	(grams)	3407 (599)	3526 (642)	3583 (555)	3580 (561)	3579 (559)
**Term birth weight**^**b**^	(grams)	3638 (425)	3670 (399)	3695 (433)	3696 (433)	3694 (433)
		n (%)	n (%)	n (%)	n (%)	n (%)
**Mother’s age** **(years)**	< 30	141 (1.8)	275 (3.5)	7 523 (94.8)	6 132 (77.2)	7 939 (100)
	30–34	57 (0.8)	193 (2.8)	6 761 (96.4)	5 851 (83.5)	7 011 (100)
	35 +	21 (0.7)	70 (2.3)	3 001 (97.8)	2 749 (88.9)	3 092 (100)
**Parity**	1	79 (1.1)	227 (3.1)	7 069 (95.6)	5 644 (76.5)	7 375 (100)
	2	86 (1.3)	204 (3.0)	6 595 (95.8)	5 807 (84.3)	6 885 (100)
	3 +	54 (1.4)	107 (2.8)	3 621 (95.7)	3 281 (86.8)	3 782 (100)
**Mother’s weight before pregnancy** **(kilograms)**	< 60	43 (1.1)	112 (2.9)	3 664 (95.9)	3 135 (82.1)	3 819 (100)
	60–69	66 (1.1)	158 (2.6)	5 808 (96.3)	4 971 (82.4)	6 032 (100)
	70–79	41 (1.1)	108 (2.9)	3 566 (96.0)	3 023 (81.4)	3 715 (100)
	80–89	27 (1.4)	76 (3.8)	1 896 (94.9)	1 602 (80.1)	1 999 (100)
	90 +	37 (1.9)	74 (3.8)	1 835 (94.3)	1 521 (78.2)	1 946 (100)
	Missing	5 (0.9)	10 (1.9)	516 (97.2)	480 (90.4)	531 (100)
**Total non-smokers**		219 (1.2)	538 (3.0)	17 285 (95.8)	14 732 (81.7)	18 042 (100)

^a^ Without restrictions on gestational length

^b^ Restricted to births at 39 to 41 weeks of gestation

**Fig. 3:**
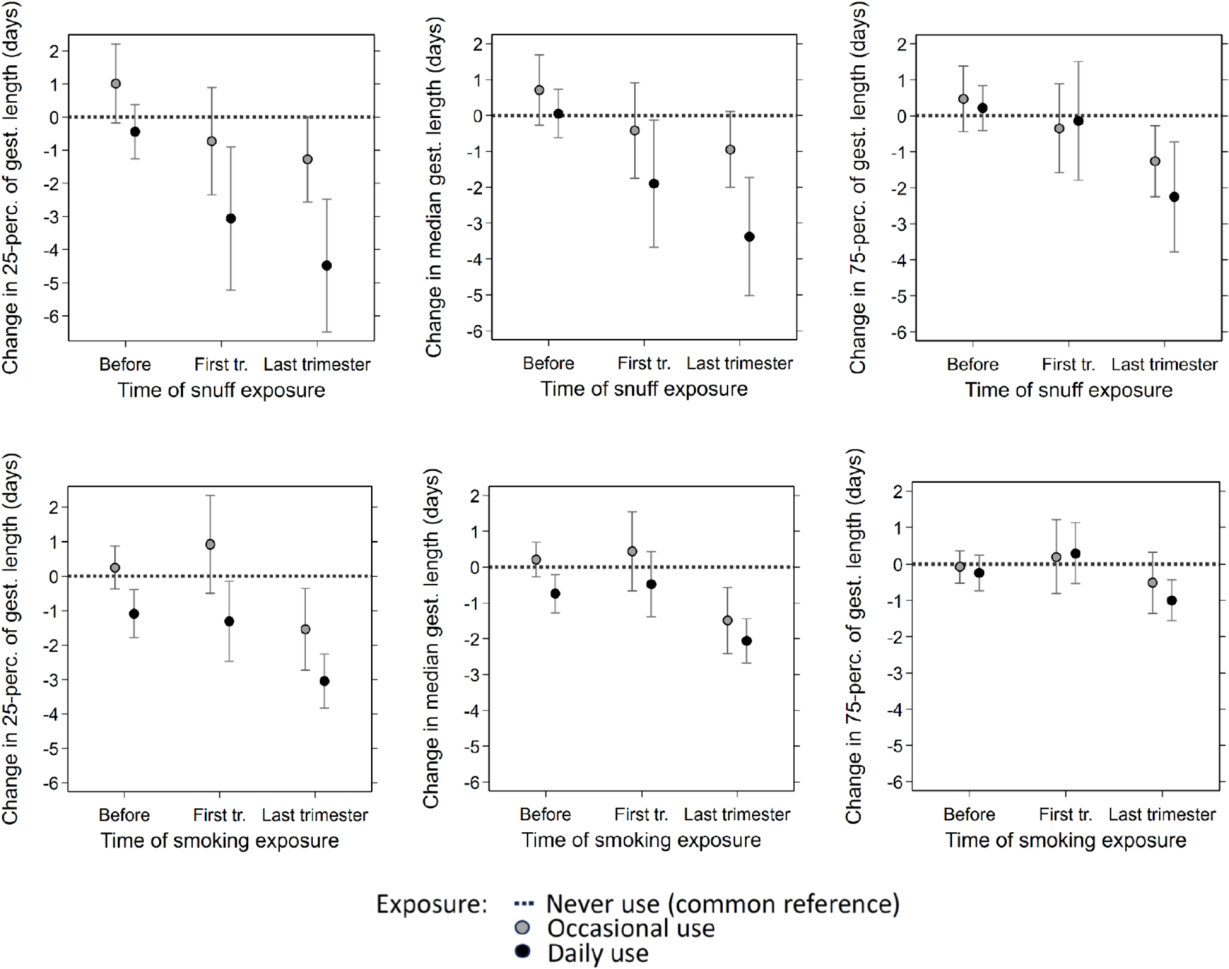
Change in 25th percentile, median and 75th percentile of gestational length by snuff use (upper panels) and smoking (lower panels) estimated by quantile regression with 95% confidence intervals. Estimation is performed jointly across time points for all exposure categories with never-users as reference (dotted line). Grey circles show estimates for occasional exposure and filled circles estimates for daily exposure at each time point

Results of the quantile regression analyses of gestational length are shown in Fig. 3. For occasional snuff use, there are only weak associations with use during any time in pregnancy. For daily snuff use, however, there was evidence of an association with use in the first trimester and especially in the last trimester. With daily use in the last trimester, the 25th percentile of gestational length was reduced by 4.5 days (95% CI: -6.5 to -2.5), the median reduced by 3.4 days (-5.0 to -1.7), and the 75th percentile reduced by 2.3 days (-3.8 to -0.7).

The estimated associations of daily cigarette smoking in the last trimester with gestational length appeared to be slightly weaker than for daily snuff use (Fig. 3), but still with substantial reductions in both the 25th percentile (-3.1 days, -3.8 to -2.3) and the median (-2.1 days, -2.7 to -1.4). The associations with gestational length were not significantly different for daily smoking and daily snuff use in the last trimester (test of difference *p*-value of 0.19, 0.14 and 0.13 respectively for the 25th percentile, the median, and the 75th percentile).

**Fig. 4:**
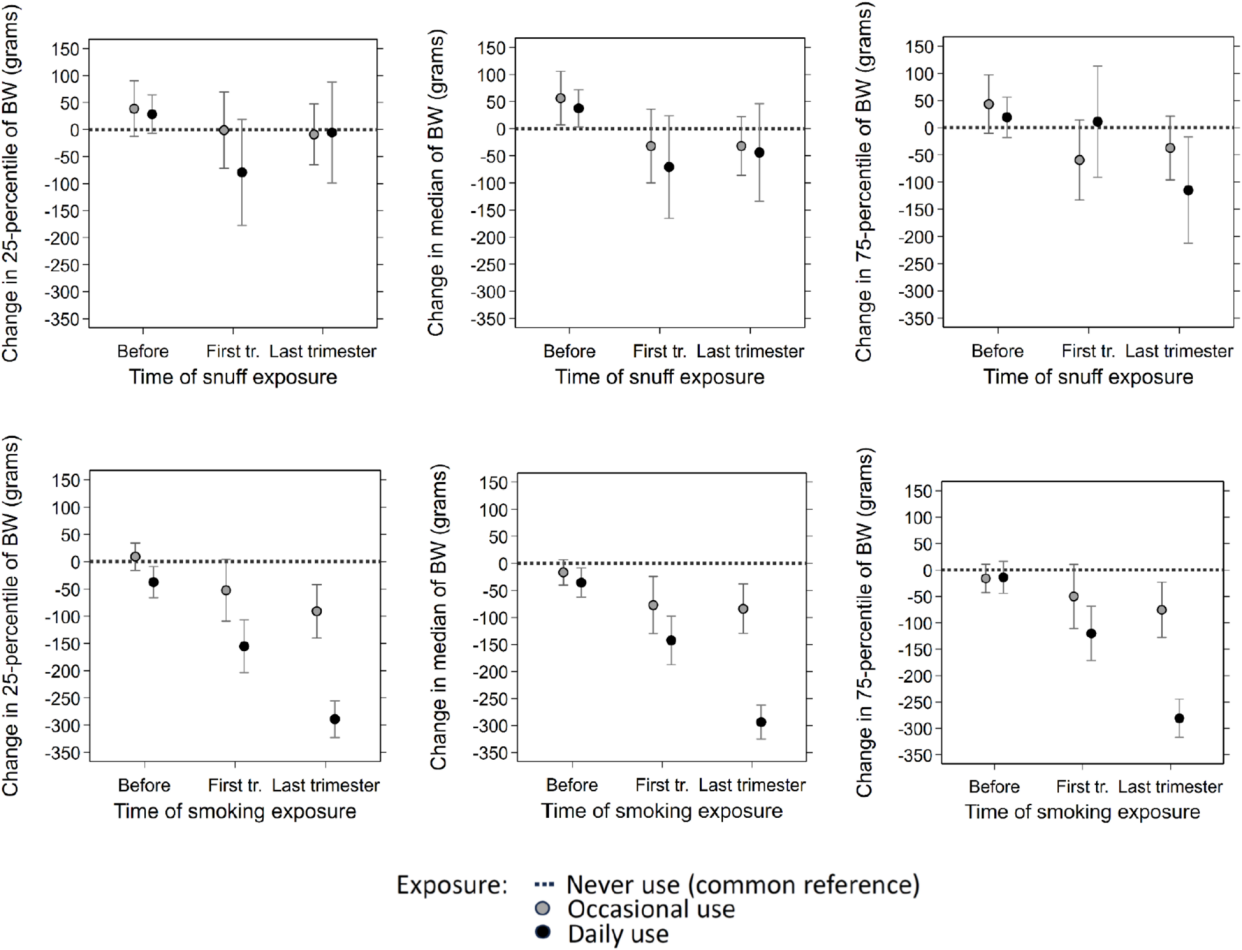
Change in 25th percentile, median and 75th percentile of birth weight for term births (39–41 weeks gestational length) by snuff use (upper panels) and smoking (lower panels) estimated by quantile regression with 95% confidence intervals. Estimation is performed jointly across time points for all exposure categories with never-users as reference (dotted line). Grey circles show estimates for occasional exposure and filled circles estimates for daily exposure at each time point

Snuff use was only weakly associated with term birth weight (39–41 weeks). Except for the 75th percentile, effect estimates with daily use were small and confidence intervals did not exclude zero (Fig. 4). The median birth weight was reduced by 44 g (-134 to 46) for daily snuff use in the last trimester. For smoking, in contrast, associations were strong with a clear dose–response pattern. Daily smoking in the last trimester reduced the median term birth weight by 294 g (95% CI: -325 to -262) and associations were similar at the 25th and the 75th percentile. The associations for daily snuff use and daily smoking with birth weight were significantly different (test of difference p-value of < 0.001, < 0.001 and 0.002 respectively for the 25th, the 50th, and the 75th percentile).

Since the strongest associations with snuff and smoking were seen during the last trimester of use, we inspected the cumulative distribution function of gestational length (left panels) and birth weight among term births (right panels) for categories of snuff use (top panels) and smoking (bottom panels) in the last trimester (Figure S2, supplementary material). For snuff use, the distribution of gestational length is markedly shifted downwards with daily use and less so with occasional use. The shift is more pronounced in the lower part of the distribution, with shorter gestation particularly from around 259 days (corresponding to week 37 of pregnancy). For birth weight among term births, there is little evidence of any difference with snuff use, except perhaps for the upper part of the distribution. The effect of smoking on term birth weight is much stronger, more uniform, and with a clearer dose–response pattern compared to snuff effects. In contrast, snuff has more apparent effects on gestational length.

Daily snuff users in the last trimester had a 1.4-fold odds of having a non-spontaneous delivery (odds ratio = 1.4, 95% CI: 1.1–1.9). The association between daily snuff use and shorter gestational length may therefore be partly mediated by clinical interventions. Excluding elective cesarean and other non-spontaneous deliveries did, however, not affect the associations with snuff use. The median gestational length was still reduced by 3.4 days with daily snuff use in the last trimester (95% CI: -5.0 to -1.8), while the 25th percentile increased from -4.5 days to -4.6 days (95% CI: -7.0 to -2.2). Those who smoked daily during the last trimester also had a 1.4-fold odds of having a non-spontaneous delivery (odds ratio = 1.4, 95% CI: 1.3–1.6). The association of daily smoking with gestational length was reduced from -2.1 to -1.4 days (95% CI: -2.0 to -0.73) after excluding non-spontaneous deliveries, while the association at the 25th percentile was reduced from -3.1 to -2.9 days (95% CI: -3.8 to -2.0).

Since more than one-third had incomplete or missing information on snuff-use (Fig. 1), we used an inverse probability weighted analyses to assess the possibility of selection bias. The probability that information on snuff-use was present was estimated from a logistic model using mother’s country of birth, her age, weight before pregnancy, smoking and marital status as predictors. The estimated associations in median gestational length and term birth weight were practically unchanged in weighted analyses, suggesting that selection may not have caused serious bias.

## Discussion

Using recent data from two regions of Norway, we find that daily snuff use throughout the third trimester was associated with a reduction in median gestational length by 3.4 days. This association with gestational length was greater than the 2.1 days reduction seen with daily cigarette smoking. We found little evidence that daily snuff use affects fetal growth, while smoking was strongly associated with reduced birth weight.

Previous studies reported that any snuff use though pregnancy increased the risk of early preterm birth 1.7-fold, increased the risk of late preterm birth 1.3-fold, and reduced mean birth weight by 73 g [9, 13–15]. Our study suggests that the strongest effects are among women who use snuff daily throughout pregnancy.

Snuff may provide a higher plasma nicotine concentration than cigarettes [8]. It is possible that the specific association of daily snuff use with shorter gestation is related to persistent and particularly high doses of nicotine. Mechanisms by which nicotine might affect gestational length are not known. If a high dose of nicotine is involved in mechanisms that trigger delivery, the effect should be seen with snuff use late in pregnancy. If persistent use throughout the pregnancy alters the fetus or conditions of the pregnancy in ways that reduce gestational length, effects should be seen with use throughout the pregnancy. Since most women who used snuff daily during the last trimester also had used snuff throughout the pregnancy, we were unable to separate these two types of effect. We can say only that use up to the first trimester but not thereafter have little or no impact on gestational length.

Daily smoking had a much stronger effect than daily snuff use on birth weight. This suggests that the predominant effect of smoking on fetal growth is not through nicotine but through the additional toxic chemicals in cigarette smoke or reduced oxygen supply to the fetus.

Among the strengths of our study is the access to population-based data that separates occasional from daily snuff use in pregnancy. The information on snuff exposure is to some degree prospectively collected. The information on birth weight and gestational length has a high degree of reliability.

Our data were limited in statistical power to address rare and important health outcomes of pregnancy such as fetal or neonatal death. We are therefore not able at this point to assess whether the reduced gestational length also corresponds with poorer infant health outcomes. It should, however, be noted that the reduction in gestational length with daily snuff use was more pronounced for the shorter gestational lengths. A second limitation is unmeasured confounding by social conditions that might distinguish snuff and non-snuff users. A third limitation is the high number lacking information of snuff use. We assessed the potential for selection bias using available variables in an IPW-analysis. Although we did not find evidence of bias, we cannot exclude the possibility of selection bias.

The recording of birth weight is expected to have high reliability in the MBRN. Errors in due date estimation at ultrasound or date of LMP, however, will result in errors in the gestational length information in the MBRN. Whether there are systematic differences in errors of the gestational length information for daily snuff users is difficult to assess, but the possibility cannot be excluded.

We compared the association of snuff dose (none, occasional and daily) with associations with similar dose data for smoking. These dose-variables may, however, not be directly comparable since the pattern of occasional and daily use may differ between snuff users and smokers. We also had the opportunity to exclude smokers in the snuff analysis but lacked information on snuff use in the data used in the smoking analysis.

The mid dose category of “occasional use” included those with unknown frequency of use and use sometimes. This should not be considered a category of specified moderate use, but rather a mixed category of use of different frequencies.

Associations between snuff use and gestational length were not attenuated when we excluded elective cesarean deliveries and other non-spontaneous deliveries, but for smoking there was a slight attenuation. Association with snuff or smoking may hypothetically be partly mediated by non-spontaneous deliveries. Such a mediating pathway may, however, include both social and biological mechanisms. Mediation due to social factors, such as a stronger tendency of the mother to seek a non-spontaneous delivery, should be removed, but it would not be correct to exclude medical interventions that might be the result of snuff use. Since we cannot distinguish these two types of interventions, we present estimates of the total association as our main analysis.

## Conclusions

Daily snuff use through the last trimester of pregnancy appeared to reduce the median gestational length by more than three days and the 25th percentile by more than four days. A reduction in already short gestational lengths may have negative effects on the baby. Daily use of snuff also reduced birth weight, but not as much as daily smoking. This suggests that the predominant effect of smoking on fetal growth is not through nicotine but through the additional toxic chemicals in cigarettes or by reduced oxygen supply to the fetus.

Our data confirm a growing body of literature showing that frequent snuff use in pregnancy represents a health concern. Our analyses have several limitations and more data with larger sample sizes and more complete information on snuff use is needed to clarify the effects on gestational length and other health outcomes.

## Supplementary Information

Supplementary Material 1.

## Data Availability

The data that support the findings of this study are available from The Medical Birth Registry of Norway but restrictions apply to the availability of these data, which were used under license for the current study, and so are not publicly available. Data are however available from the authors upon reasonable request and with permission of The Medical Birth Registry of Norway.
