# Supplementary material for: Daily snuff use during pregnancy, gestational length and birth weight; register-based study
Source: BMC Pregnancy Childbirth. 2024 Oct 4;24:646. doi: 10.1186/s12884-024-06863-8 (PMC11450978; doi:10.1186/s12884-024-06863-8)
Supplement: Supplementary file 1 — Supplementary Material 1. [file 12884_2024_6863_MOESM1_ESM.docx]

**Supplementary information**

**Table S1. Frequency of combined use of snuff and smoking, west and central region on Norway, 2020-2021**

|  |  | **SNUFF use in last trimester** | | |  |  |
| --- | --- | --- | --- | --- | --- | --- |
|  |  | No use  n (%) | Occasional n (%) | Daily use  n (%) |  | Total  n (%) |
| **Smoking in last**  **trimester** | No | 17 911 (97.3) | 262 (1.4) | 245 (1.3) |  | 18 418 (100) |
|  | Occasional | 67 (16.8) | 326 (81.9) | 5 (1.3) |  | 398 (100) |
|  | Daily | 118 (90.8) | 3 (3.1) | 9 (6.9) |  | 130 (100) |

**Table S2. Descriptive information of key variables by categories of smoking, Norway 2018-2021**

|  |  | **Smoking in last trimester** | | | **Never** |  |
| --- | --- | --- | --- | --- | --- | --- |
|  |  | **Daily smoke** | **Occasional** | **No smoking** | **smoking** | **Total** |
|  |  | Mean (SD) | Mean (SD) | Mean (SD) | Mean (SD) | Mean (SD) |
| **Gestational length** | (days) | 275.6 (15.4) | 276.0 (18.4) | 278.5 (13.3) | 278.5 (13.3) | 277.2 (18.8) |
| **Birth weight**^†^ | (grams) | 3291 (597) | 3453 (654) | 3570 (557) | 3571 (556) | 3558 (570) |
| **Term birth weight^‡^** | (grams) | 3471 (439) | 3607 (451) | 3684 (440) | 3685 (439) | 3680 (440) |
|  |  |  |  |  |  |  |
|  |  | n (%) | n (%) | n (%) |  | n (%) |
| **Mother’s age**  **(years)** | <30 | 671 (1.2) | 337 (0.6) | 55 341 (98.2) | 52 616 (93.4) | 56 349 (100) |
|  | 30-34 | 590 (1.2) | 251 (0.5) | 49 093 (98.3) | 47 054 (94.2) | 49 934 (100) |
|  | 35+ | 440 (1.8) | 141 (0.6) | 24 292 (97.7) | 22 908 (92.1) | 24 873 (100) |
|  |  |  |  |  |  |  |
| **Parity** | 1 | 472 (0.8) | 291 (0.5) | 57 053 (98.7) | 53 882 (93.2) | 57 816 (100) |
|  | 2 | 589 (1.2) | 265 (0.5) | 48 892 (98.3) | 47 014 (94.5) | 49 746 (100) |
|  | 3+ | 640 (2.7) | 173 (0.7) | 22 781 (96.6) | 21 682 (91.9) | 23 594 (100) |
|  |  |  |  |  |  |  |
| **Mother’s weight before pregnancy** | <60 | 332 (1.2) | 160 (0.6) | 27 911 (98.3) | 26 762 (94.2) | 28 403 (100) |
|  | 60-69 | 390 (0.9) | 211 (0.5) | 41 392 (98.6) | 39 728 (94.6) | 41 993 (100) |
|  | 70-79 | 301 (1.2) | 131 (0.5) | 24 703 (98.3) | 23 381 (93.0) | 25 135 (100) |
|  | 80-89 | 235 (1.8) | 88 (0.7) | 13 004 (97.6) | 12 219 (91.7) | 13 327 (100) |
|  | 90+ | 314 (2.4) | 101 (0.8) | 12 647 (98.2) | 11 756 (90.0) | 13 062 (100) |
|  | Missing | 129 (1.4) | 38 (0.4) | 9 069 (98.2) | 8 732 (94.5) | 9 236 (100) |
|  |  |  |  |  |  |  |
| **Total** |  | 1 701 (1.3) | 729 (0.6) | 128 726 (98.2) | 122 578 (93.5) | 131 156 (100) |
|  |  |  |  |  |  |  |

† Without restrictions on gestational length

‡ Restricted to births at 39 to 41 weeks of gestation


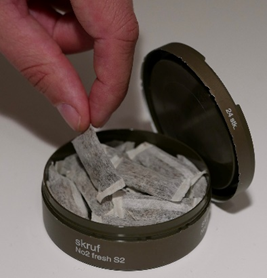


**Figure S1.**

Swedish snus, or snuff, is typically packed in pouches that are placed under the upper lip.

**Figure S2.**

Cumulative distribution of gestational length and birth weight for categories of snuff use in the last trimester (upper panels) and cumulative distribution of gestational length and birth weight for categories of smoking in the last trimester (lower panels). Horizontal dotted lines show the 25^th^ percentile, the median and the 75^th^ percentile. Only births between 39 and 41 weeks of gestation were included in the distribution of birth weight.
